# Smartphone-integrated portable microfluidic platform for liver biomarker quantification using deep learning

**DOI:** 10.1038/s41598-025-29431-2

**Published:** 2025-11-21

**Authors:** Neha Ingole, Sangeeta Palekar, Madhusudan B. Kulkarni, Jayu Kalambe, Richa Khandelwal

**Affiliations:** 1 Department of Electronics Engineering, Shri Ramdeobaba College of Engineering and Management, Nagpur, 440013 Maharashtra India; 2 Department of Electronics and Communication Engineering, Manipal Institute of Technology, Manipal Academy of Higher Education (MAHE), Manipal, 576104 Karnataka India; 3 Department of Electronics Engineering, Ramdeobaba University, Nagpur, 440013 Maharashtra India

**Keywords:** Liver biomarkers, Smartphone-based sensing, Deep learning, Microfluidics, Point of care testing, Biological techniques, Biomarkers, Computational biology and bioinformatics, Engineering, Health care

## Abstract

Accurate and decentralized liver biomarker testing is critical for early diagnosis and monitoring of hepatic dysfunctions, particularly in resource-constrained settings. This work presents a novel smartphone-integrated colorimetric sensing platform that combines microfluidics, deep learning, and mobile health technologies to estimate liver biomarkers quantitatively. A stereolithography (SLA) 3D-printed microfluidic flow cell, optimized for low reagent use and high optical clarity, processes 100 µL of sample-reagent mixture via a peristaltic pump at 50 µL/s. Biomarker-specific chromogenic reactions are imaged within a controlled lighting enclosure using multiple smartphone models and analyzed using a convolutional neural network (CNN) for a regression approach. The system achieves clinically relevant detection ranges of 0.1–20 mg/dL for direct and total bilirubin, and 10–300 U/L for alanine aminotransferase (ALT) and aspartate aminotransferase (AST), with limits of detection of 0.1 mg/dL, 0.05 mg/dL, 2.97 U/L, and 2.5 U/L, respectively. A two-point smartphone adaptability framework ensures robust cross-device performance without retraining. An Android application has been developed, which provides users with disease identification, real-time inference, and visualization of result. This clinical-grade analyzer features an average coefficient of determination (R^2^) of 0.997 for all biomarkers, and the repeatability is shown by coefficients of variation under 3%. This innovative, cost-effective and portable solution gives precise liver function assessment, making it ideal for rural healthcare and mobile diagnostics.

## Introduction

Liver conditions like hepatitis, cirrhosis, and hepatocellular carcinoma are challenging for global health. According to the World Health Organization, over two million people die each year due to liver-related issues. This is due to delay in diagnosis and lack of healthcare systems. Prompt detection of disease and regular monitoring of liver health is important for on time treatment and better results^1,2^. Regular monitoring of liver function is done by analysing biochemical indicators such as total and direct bilirubin, ALT, and AST levels. These indicators give useful information about the hepatocellular damage, cholestasis, and hepatic metabolism, which is an important tool for clinical decision-making in both acute and chronic liver conditions. Standard laboratory instruments, such as automated spectrophotometric techniques, are the reference point due to their precision and consistency. But due to their availability only in labs and hight cost and the need for trained personnel to operate it creates obstacles in remote and under-resourced healthcare services^3^.

Multiple biosensing techniques have been studied for biochemical analysis to address these deficiencies. The analysis done with colorimetric uses chromogenic reactions to produce analyte-dependent color transformations. This analysis has proven to be the easiest method due to its chemistry and compatibility with existing reagents. Laboratory spectrophotometers or UV–Vis analyzers, which are highly accurate, are some traditional approaches, but they are challenging due to their portability. Due to the disadvantages of all these methods, new POCT strategies were developed. These new methods include dry chemistry strips, portable spectrophotometers, and microfluidic test kits. Qualitative or semi-quantitative results for glucose or creatinine were obtained using Strip-based techniques. But these techniques are not fully accurate for liver biomarkers. Meanwhile, these portable spectrophotometers are found to be expensive and also, they are technically complex for multiple use^3,4^. On the other hand, in electrochemical tests for liver biomarkers are having some demerits, like contamination of electrode, surface instability, some drifts in the measurement and calibration, and blood serum and plasma interference^5,6^. Centralized immunoassay frameworks use Chemiluminescence^7^ and electrochemiluminescence (ECL)^8^, but they are not portable due to some of their reagent and environmental factors^9,10^. Similarly, Fluorescence-based detection is used for multiplexing, but some factors, like photobleaching, degradation of the probe, and intricate optics, do not allow portability of the device^11^.

Simultaneously, the potential of smartphones as a tool for diagnosis has increased due to recent advancements in mobile health analysis. The images that are taken with high-resolution, easy-to-operate interfaces, and wireless connectivity of smartphones increase the portability and compactness of the device^12,13^. Measuring glucose, cholesterol, and biomarkers associated with infectious diseases can be done with these devices. But these technologies are not suitable in clinical settings due to some limitations, like varying lighting conditions and differences in the pixel size of cameras. Most existing studies rely on basic RGB value extraction, which interprets spatial characteristics of colorimetric reactions, thereby reduces sensitivity and restricts adaptability across different smartphone models^12–15^.

This research demonstrates a smartphone-based microfluidic system for testing liver biomarkers, which is focused on overcoming these challenges. A microfluidic flow cell is used in the system, which is made of stereolithography (SLA), developed for increasing optical clarity and using minimal reagents. A specialized casing with evenly distributed diffused LED lighting is used to place the flow cell. This is done to minimize the environmental effects and give stable lightning conditions. The smartphone camera is used to capture the images, and then the images are analyzed using a convolutional neural network (CNN) regression model. A non-linear spectral-spatial characteristic is identified using this framework, which is not supported in traditional RGB parameters. In comparison with the traditional laboratory analyzers, this developed system is lightweight, low cost, and can be operated without any special training. This approach is capable of detecting liver disease in areas with limited resources where there is a critical need for this device.

## Materials and methods

### Chemicals and consumables

The direct bilirubin reagent (RD) was prepared by using Sulfanilic acid having 29 mmol/L of concentration, hydrochloric acid at 0.24 mol/L, and sodium nitrite at 11.6 mmol/L. The total bilirubin reagent (RT) had the same base composition with added Duposol^®^ (3% w/v) as a solubilizing agent to facilitate measurement of total bilirubin. A bilirubin calibrator containing freeze-dried bilirubin in a protein matrix was used for calibration and standard curve preparation^16^.

The ALT reagent is made from a substrate reagent R1 containing L-alanine (220 mmol/L) and 2-oxoglutarate (12 mmol/L) as the primary enzymatic reaction components. The color reagent R2 comprised 2,4-dinitrophenylhydrazine (2,4-DNPH, 7 mmol/L), which forms a colored hydrazone with pyruvate. A calibrator solution containing sodium pyruvate with a concentration of 170 U/L was used for standardization. An alkaline reagent with sodium hydroxide (4 N) was also employed to develop the final color for quantification^17^.

The AST reagent kit comprises four key components. The Substrate Reagent (R1) consists of L-Aspartic Acid (120 mmol/L) and 2-Oxoglutarate (12 mmol/L), which are necessary for starting the enzymatic reaction. 2,4-Dinitrophenylhydrazine (2,4-DNPH) with a concentration of 6 mmol/L are included in the Color Reagent (R2), that forms a chromogenic complex with oxaloacetate. An additional Alkaline Reagent (R3), composed of Sodium Hydroxide (4 N), is concentrated at 1:10 ratio with deionized (DI) water before using it. The Calibrator is prepared using Sodium Pyruvate with an activity of 160 U/L, enabling accurate quantification of SGOT (AST) activity^18^. All the chemical reagents and calibrators are procured from Avecon Healthcare Pvt. Ltd, India. DI water is purchased from Emplura, India. All the electronics components, like the motor driver L298N, 12 V peristaltic pump, 5 V diffused LED strip, and ESP32 microcontroller unit, were purchased from robu.in.

### Sample preparation

Samples were prepared using commercially available colorimetric reagent kits. Sample and reagent were mixed and incubated externally at 37 °C for 5 min in a calibrated incubator to allow the enzymatic reaction to proceed to the recommended endpoint. After this incubation period the reacted sample was aspirated into the microfluidic flow cell for imaging.

#### Total and direct bilirubin assay

The bilirubin assay is based on the diazo reaction, in which bilirubin forms a colored complex known as azobilirubin upon reacting with diazotized sulfanilic acid. In acidic conditions, sulfanilic acid (C₆H₇NO₃S) and sodium nitrite (NaNO₂) react to produce a diazonium salt, which couples with bilirubin to generate a pinkish chromophore.

$${\text{Diazotization: Sulfanilic acid }}+{\text{ Sodium nitrite}} \to {\text{Diazotized Sulfanilic Acid}}$$

$${\text{Colorimetric reaction}}:{\text{Bilirubin }}+{\text{ Diazotized Sulfanilic Acid}} \to {\text{Azobilirubin }}\left( {{\text{pinkish color}}} \right)$$

For total bilirubin estimation, the reagent mixture contains a solubilizing agent—Duposol^®^ (3% w/v)—which helps displace unconjugated (indirect) bilirubin from albumin, allowing both conjugated and unconjugated bilirubin to react. For calculating the direct bilirubin, the reagent is insoluble, therefore only direct bilirubin takes part in the reaction. Due to this selective reactivity, it promotes separation between total and direct bilirubin fractions. The working reagent is developed by combining 1 mL of sodium nitrite (RN) with 4 mL of RT (for total bilirubin) or RD (for direct bilirubin). For each test, 5 µL of test sample is added to 100 µL of the working reagent and inserted into a microfluidic flow cell designed for colorimetric sensing.

#### ALT (alanine aminotransferase/SGPT) assay

The ALT (SGPT) sensing is having a two-step enzymatic reaction. First, ALT (SGPT) enables the aminotransferases between L-alanine and 2-oxoglutarate (α-ketoglutarate), producing pyruvate and glutamate. In the second step, the pyruvate interacts with 2,4-dinitrophenylhydrazine (2,4-DNPH) which forms a brownish-red hydrazone complex under alkaline conditions^19^. The power of this colored complex is equivalent to the ALT concentration.

$${\text{Enzymatic reaction:}}\,\,{\text{L-Alanine }}+{\text{ 2-Oxoglutarate}} \to {\mathrm{SGPTPyruvate}}\,+\,{\mathrm{Glutamate}}$$

$${\text{Colorimetric reaction:Pyruvate }}+{\text{ }}2,4{\mathrm{-DNPH}} \to {\text{Brownish red hydrazone complex}}$$

To prepared the working reagent it is mixed as 1 part of the color reagent (R2) with 4 parts of the substrate reagent (R1). Each test is included with the addition of 5 µL of serum to 100 µL of the working reagent, which then enters into the microfluidic flow cell for image capturing.

#### AST (aspartate aminotransferase/SGOT) assay

The evaluation of Aspartate Aminotransferase (AST), is done using the Modified Reitman and Frankel’s Colorimetric 2,4-DNPH method. AST enables the changeable transamination of L-aspartate and 2-oxoglutarate, giving oxaloacetate and L-glutamate. The oxaloacetate which is produced reacts with 2,4-dinitrophenylhydrazine (2,4-DNPH) forming a brownish-red hydrazone complex under alkaline conditions. The generated color intensity corresponds directly with AST concentration.

$${\text{Enzymatic reaction:L-Aspartate}}\,+\,{\mathrm{2-Oxoglutarate}} \to {\mathrm{Oxaloacetate}}\,+\,{\mathrm{L-Glutamate}}$$

$${\text{Colorimetric reaction: Oxaloacetate}}\,+\,{\mathrm{2}},{\mathrm{4-DNPH}} \to {\text{Brownish-red hydrazone complex}}$$

For preparing the working reagent, it is mixed with Reagent 1 (R1), Reagent 2 (R2), and pre-diluted Alkaline Reagent (R3) in a 1:1:6 ratio. Before this process, deionized water was used to dilute R3 in a 1:10 ratio (one part R3 with nine parts deionized water) to obtain the required dilution for working. 5 µL of the serum sample is added to 100 µL of the freshly prepared working reagent for each test. After sample preparation, which is prepared by mixing the working reagent and the test sample, the resultant sample is suctioned into a microfluidic flow cell for colorimetric sensing.

### Experimental setup for smartphone-based sensing platform

**Fig. 1:**
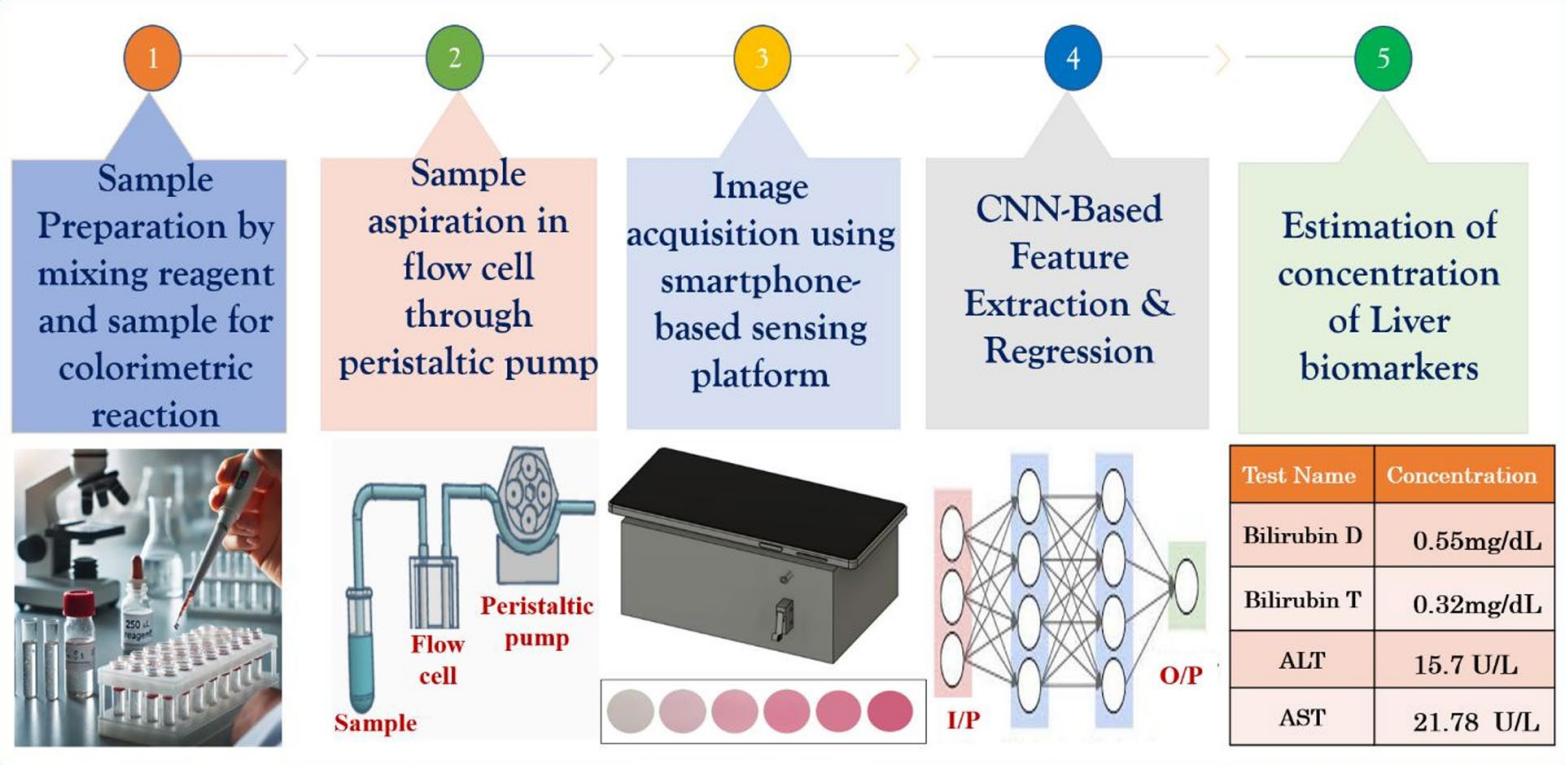
Liver biomarker system and its complete process flow.

The proposed methodology combines colorimetric biochemical assays with image-based analysis to identify liver biomarkers. The coloured chemical reactions are the main factor on which the biomarker analyzer depends. In this, the colorimetric variation intensity is proportional to analyte concentration. After this process, the test sample is suctioned from the microfluidic flow cell. This is done by using a peristaltic pump. This process makes sure that there is controlled flow and consistent reagent sample contact. The optically transparent chamber of the flow cell confirms that the uniform transmission of light is achieved, and the resulting images of the colorimetric changes are captured using a smartphone camera. Then CNN is used to process the images. The prediction of the biomarker concentrations without depending on traditional RGB intensity extraction is done using a CNN. Figure 1 shows the workflow that highlights the combination of microfluidics, smartphone-based imaging, and AI-enabled analysis, which gives a complete, efficient, and portable testing platform.

**Fig. 2:**
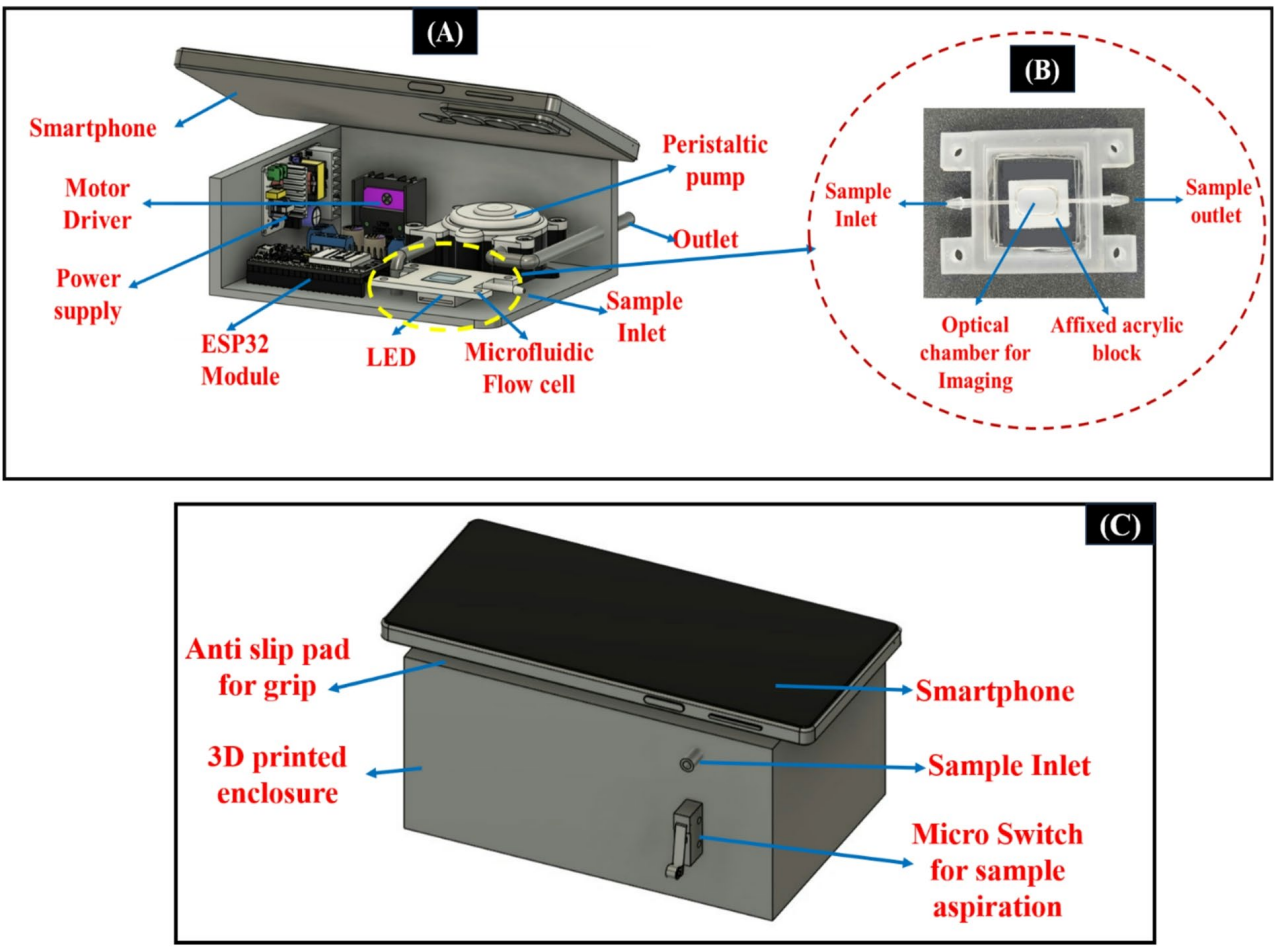
(**A**) Smartphone-based sensing platform with the integration of the ESP32 controller, motor driver, peristaltic pump, flow cell, and smartphone interface inside a light-isolated 3D-printed enclosure. (**B**) SLA-fabricated flow cell developed using optically clear resin for imaging. (**C**) 3D printed enclosed device having a micro switch for sample aspiration.

A 3D-printed microfluidic flow cell is developed, which provides a uniform illumination and an electronic control unit, as shown in Fig. 2A. The fabrication of the microfluidic flow cell is done using stereolithography (SLA) 3D printing, as illustrated in Fig. 2B. It is designed to hold the test sample within a fixed path. The flow cell is placed directly below the smartphone camera for capturing high-resolution images of the developed colorimetric reaction. An LED strip of 5 V was mounted within the setup for uniform illumination. A custom-designed black 3D-printed enclosure is used to mount the whole assembly, as shown in Fig. 2C.

The user needs to press the microswitch to begin aspiration after the reagent is prepared and the sample is mixed. For achieving the sample aspiration, a peristaltic pump operates at a calibrated flow rate of 50 µL/s. Once the sample reaches the flow cell, the smartphone automatically captures the image. Then the image is processed through a trained CNN for biomarker prediction. The developed flow cell is single-channelled, which enables one assay to be performed per test cycle. After the image is captured, the system automatically dispenses the test sample within 2 s using the same peristaltic pump. After each test cycle, the flow cell is rinsed with deionized (DI) water. This is done to prevent sample-to-sample cross-contamination. The microfluidic design ensures complete evacuation of the previous sample and maintains an air gap between consecutive tests, thereby preventing sample mixing. The pump, along with the LED lighting module, is controlled via an ESP32 microcontroller unit.

All the colorimetric images needed for analysis were acquired using the smartphone camera in manual mode. The ISO, shutter speed, and white balance were fixed at 100, 1/60 s, and daylight mode, respectively. Automatic functions, including auto-exposure, auto-white-balance, HDR, and tone-mapping, were disabled. This was done to avoid dynamic adjustment during capture. The imaging setup was placed within a light-isolated 3D-printed chamber illuminated by a constant-intensity LED source. To avoid any deviation beyond the acceptable threshold and triggering recalibration, the LED output was verified at the beginning of each session by recording the reference signal of deionized water.

### Design and fabrication of the microfluidic flow cell

**Fig. 3:**
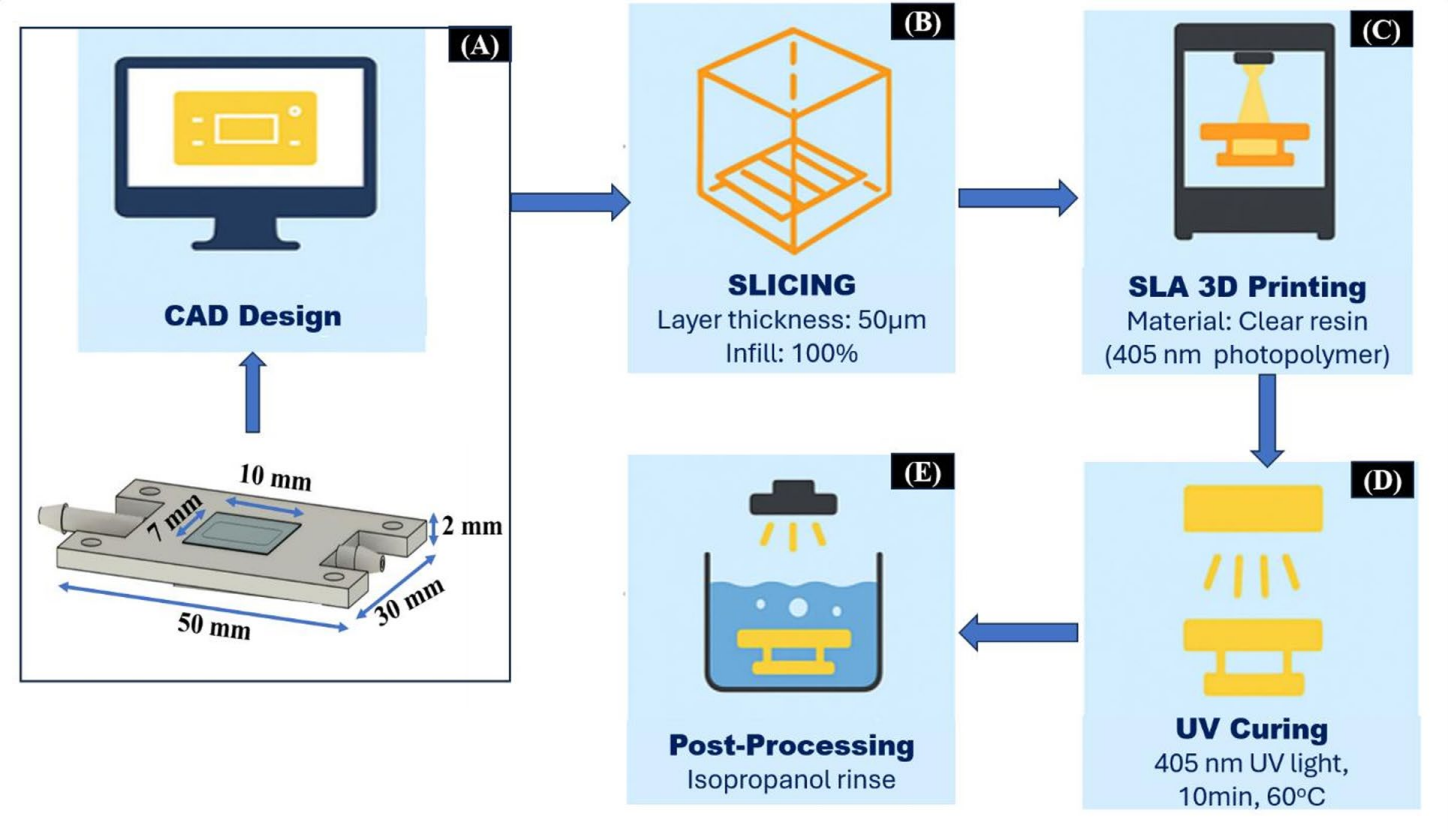
Fabrication workflow of the SLA-printed microfluidic flow cell. (**A**) CAD design with dimensions. (**B**) Digital slicing of the STL file in the PreForm software having 50 μm resolution. (**C**) SLA printing has transparent resin for high-fidelity channel structures. (**D**) Post-processing, along with IPA washing to remove residual resin. (**E**) UV curing at 405 nm for improving transparency and mechanical stability.

To ensure a high level of transparency, low surface roughness, and minimize optical distortion the microfluidic flow cell was fabricated with optically clear resin for accurate color capture as illustrated in Fig. 3. Figure 3A shows the microfluidic geometry, including the inlet–outlet ports, optical chamber, and channel layout. It is designed using SolidWorks CAD software. The design was refined for laminar flow and kept aligned with the smartphone camera’s focal plane for precise imaging. In the next step (Fig. 3B), the CAD model was exported in STL format, and it was digitally sliced using PreForm software at a resolution of 50 μm.

As shown in Fig. 3C, the device was fabricated on a commercial SLA 3D printer using optically transparent photopolymer resin. After the printing process is completed, the device underwent post-processing (Fig. 3D), where it is immersed in isopropyl alcohol (IPA) for the purpose of removing uncured resin. This process was followed by ultrasonic cleaning to ensure internal channel purity. Finally, the printed device went through a controlled UV post-curing step (Fig. 3E) at 405 nm for 30 min. This treatment is necessary to improve the resin’s crosslinking density, mechanical strength improvement and resistance to chemical.

The center of the inlet and outlet ports of the microfluidic flow cell has an optically accessible chamber (10 mm × 7 mm × 2 mm) as shown in Fig. 3(A). This device can be operated with a standard smartphone camera with an overall dimension of 50 mm × 30 mm. The ports are set up for easy combination of tubing, which enables controlled reagent and sample injection. The chamber is placed in alignment with the smartphone camera’s focal plane. This is done to capture high-quality images. The optical chamber is placed on top of the small acrylic block, which acts as a support to reduce stray light scattering and provides a uniform background. This improves the contrast during image capture.

Selecting the microfluidic flow cell minimizes sample and reagent volumes and provides a fixed optical path with controlled reagent–sample residence time, automated aspiration, and DI-wash cycles for reducing operator variability and carryover. Although reagent/sample mixing is performed off-chip in this prototype to simplify reagent handling, the flow cell ensures precise metering and residence time via the calibrated peristaltic aspiration and a fixed optical chamber geometry, which reduces operator-dependent variability in sample thickness and optical path.

## Results and discussion

### Experimental dataset generation for model training

A large number and variety of datasets are needed for running a deep learning model, which should accurately detect liver function biomarkers from colorimetric images. An experimental dataset is generated in this work, which is used to train a CNN-based regression model for biomarker concentration estimation.

Commercially available gold-standard reagents are used to prepare the dataset. These datasets ensure batch consistency and traceability in line with clinical testing protocols. A reagent is used to dilute each calibrator, which was used as the solvent during preparation. For each target analyte, six clinically relevant concentration levels were selected to encompass both normal physiological and pathological ranges. This range selection helps the model to learn discriminative features across the diagnostic range. The preparation of the sample was done by mixing reagent-analyte, and a custom-designed, stereolithography (SLA)-fabricated microfluidic flow cell using a peristaltic pump which operates at a controlled flow rate. The image was taken using a Samsung Galaxy M51 smartphone positioned 6 cm above the flow cell within a black, 3D-printed enclosure. 250 original images were captured for each concentration level. Then, 500 images per level were generated using a data augmentation technique. In total, per biomarker, 1500 original images and 3000 augmented images were generated. All images were labeled using the corresponding concentration values. This made the dataset suitable for supervised regression tasks. An overview of the dataset summary is illustrated in Table 1.

**Table 1:** Dataset summary for each liver biomarker used in deep learning model training.

Biomarker	Concentration levels	No. of original images	No. of augmented images	Total images per biomarker
Bilirubin mg/dL (direct)	0.1, 1, 5, 10, 15, 20	1500	3000	4500
Bilirubin mg/dL (total)	0.1, 1, 5, 10, 15, 20	1500	3000	4500
ALT U/L	10, 50, 100, 150, 200, 300	1500	3000	4500
AST U/L	10, 50, 100, 150, 200, 300	1500	3000	4500

### Deep learning model architecture and implementation

A CNN architecture with a regression output layer is implemented to analyze colorimetric assay images captured from the microfluidic device and to estimate biomarker concentrations relevant to liver functionality. This deep learning model is designed to predict biomarker concentrations from RGB images captured using the developed smartphone-based sensing platform.

The input given to the model is the images acquired by the smartphone camera positioned above the microfluidic flow cell. The images were resized to 128 × 128 pixels with three color channels (RGB), forming the input layer of shape 128 × 128 × 3 to preserve the color features critical for concentration estimation. The architecture of the model includes a series of convolutional layers that extract spatial and color-dependent features relevant to concentration variations. 32 filters with a 3 × 3 kernel and ReLU activation that are present in the Conv2D layer are used to detect low-level color and edge features. A 2 × 2 max-pooling layer is then employed to downsample the feature maps. Subsequent convolutional layers (with 64 and two sets of 128 filters) capture higher-level and more abstract patterns, which correspond to colorimetric shifts resulting from varying biomarker concentrations. A max-pooling operation succeeds every convolutional layer to retain dominant features and suppress noise, which is particularly useful for standardizing output for varying models of smartphones and lighting conditions, despite the enclosure and constant lighting. The CNN model architecture is given in Table 2.

**Table 2:** Architecture of the proposed CNN model.

Layer type	Configuration	Function
Input	128 × 128 × 3 (RGB image)	Encoded sample image captured from the flow cell
Conv2D	32 filters, 3 × 3 kernel, ReLU activation	Detects initial color features linked to reagent-sample interactions
MaxPooling2D	2 × 2	Reduces dimensionality, retains dominant features
Conv2D	64 filters, 3 × 3 kernel, ReLU activation	Captures intermediate patterns in color variations
MaxPooling2D	2 × 2	Prevents overfitting, compresses information
Conv2D	128 filters, 3 × 3 kernel, ReLU activation	Learns high-level biomarker-specific features
MaxPooling2D	2 × 2	Further reduces noise and improves generalization
Conv2D	128 filters, 3 × 3 kernel, ReLU activation	Reinforces key differentiators for regression
MaxPooling2D	2 × 2	Balances feature complexity and efficiency
Flatten	—	Converts 2D feature maps into 1D input for dense layer
Dense	512 units, ReLU activation	Learns nonlinear relations between features and concentration levels
Output (Dense)	1 unit, Linear activation (regression output)	Predicts biomarker concentration from image data

After flattening, the feature maps are fed into a dense layer of 512 ReLU-activated neurons, enabling the model to learn complex nonlinear relationships between color patterns and their corresponding concentrations. The architecture ends with an output layer of one neuron activated linearly, suited for continuous-value regression work, delivering predicted biomarker concentrations directly from input images. The CNN model was trained using the Adam optimizer with a learning rate of 0.001 and mean squared error (MSE) as the loss function. The standardized image dataset acquired using the device was used to generate the training and testing datasets. Further, TensorFlow and Keras frameworks were used for preprocessing and model development.

100 epochs were used to train the model, and the 40th epoch gave optimal performance. The model’s learning performance over 40 training epochs is illustrated in the graph. It is calculated using the Mean Squared Error (MSE) as the loss function. The robust regression accuracy, achieving a mean absolute error (MAE) of 4.741 and a root mean squared error (RMSE) of 7.237, and a coefficient of determination (R^2^) of 0.997 of the model was demonstrated. This indicates excellent agreement between predicted and actual biomarker concentrations^20^.

**Fig. 4:**
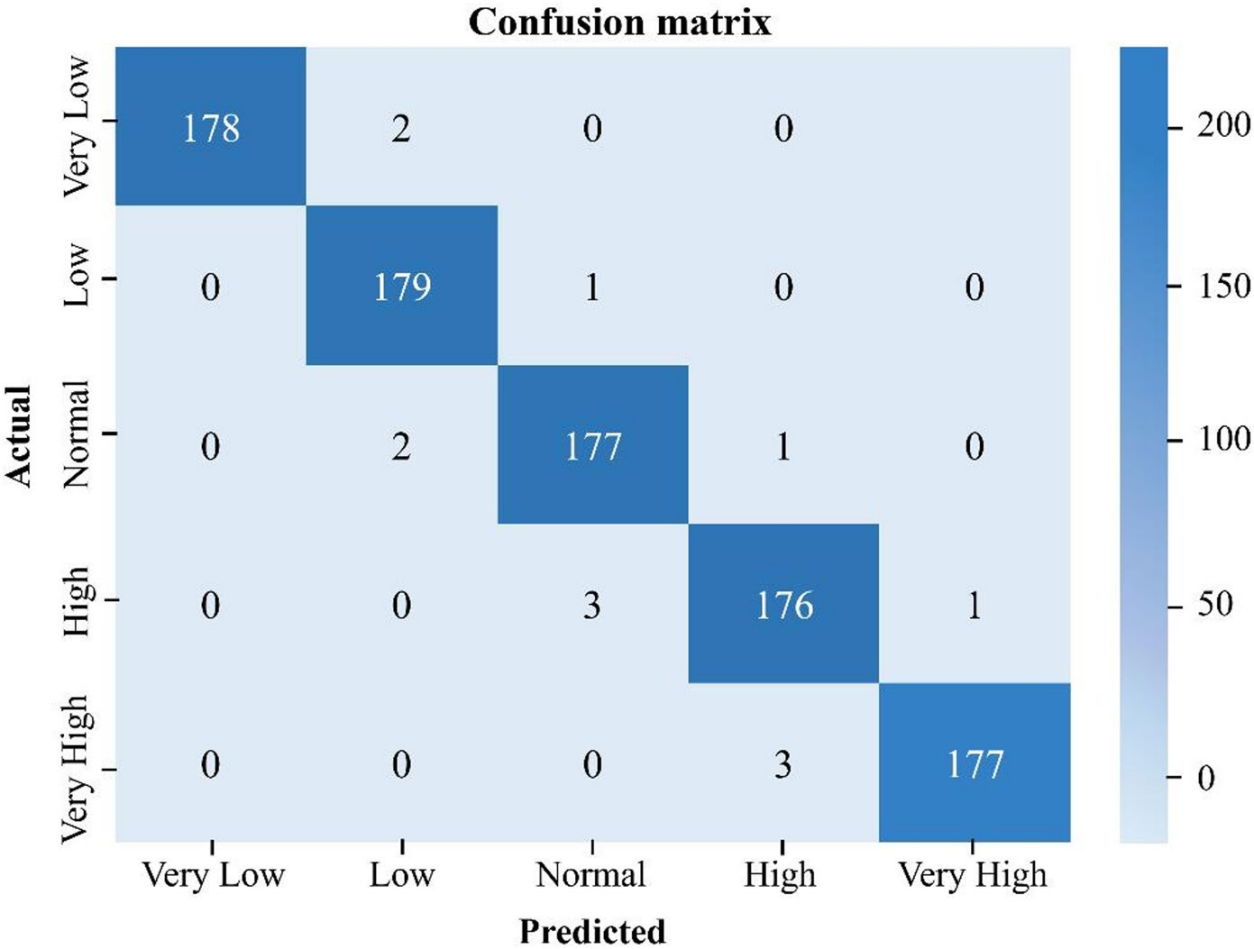
Confusion matrix for CNN model trained and tested with 900 images per biomarker. The strong diagonal dominance indicates high accuracy.

The classification performance across Total Bilirubin biomarker concentration ranges was further validated using a confusion matrix as illustrated in Fig. 4. A total of 900 images per biomarker, consists of the dataset which were evenly distributed across five clinically relevant categories: Very Low, Low, Normal, High, and Very High (∼180 images per category). The dataset was divided into training and testing subsets in an 80:20 split, with 720 images used for training and 180 images (36 per category) used for testing. Each cell represents the number of test samples for a given class that was predicted. At the same time, the diagonal dominance indicates high classification accuracy.

**Fig. 5:**
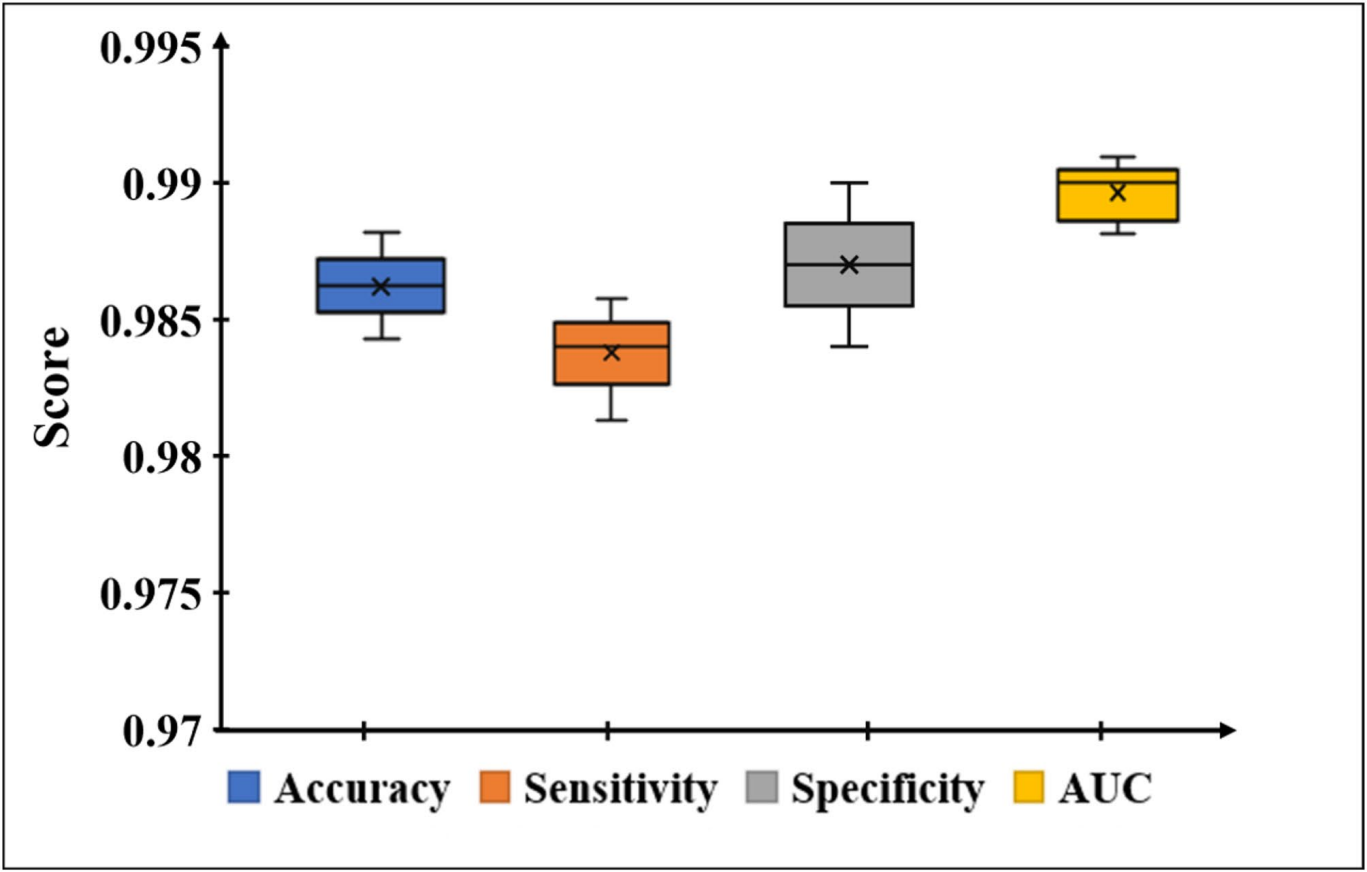
Representation of the Box plot of performance metrics averaged across all biomarkers, highlighting consistently high values with minimal variability.

The overall performance of the proposed smartphone-based sensing system across all four liver function biomarkers is shown in Fig. 5. The box plot shows the distribution of Accuracy, Sensitivity, Specificity, and Area Under the Receiver Operating Characteristic Curve (AUC) values, which is calculated as the mean across all biomarkers using equations $\:Accuracy=\frac{TP+TN}{TP+TN+FP+FN}$, $\:Sensitivity=\:\frac{TP}{TP+FN},\:$ $\:Specificity=\:\frac{TN}{TN+FP}$, where *TP*, *TN*, *FP*, and *FN* represent true positives, true negatives, false positives, and false negatives, respectively. The Area Under the Curve (AUC) was derived from the Receiver Operating Characteristic (ROC) curve. These findings are the evidence for the consistently high classification metrics, with accuracy (0.986 ± 0.002), sensitivity (0.984 ± 0.003), specificity (0.987 ± 0.003), and AUC (0.990 ± 0.001). Together, these findings validate the reliability of the proposed platform for accurate clinical decision in liver function testing.

### Smartphone variability handling using a scalable recalibration method

**Fig. 6:**
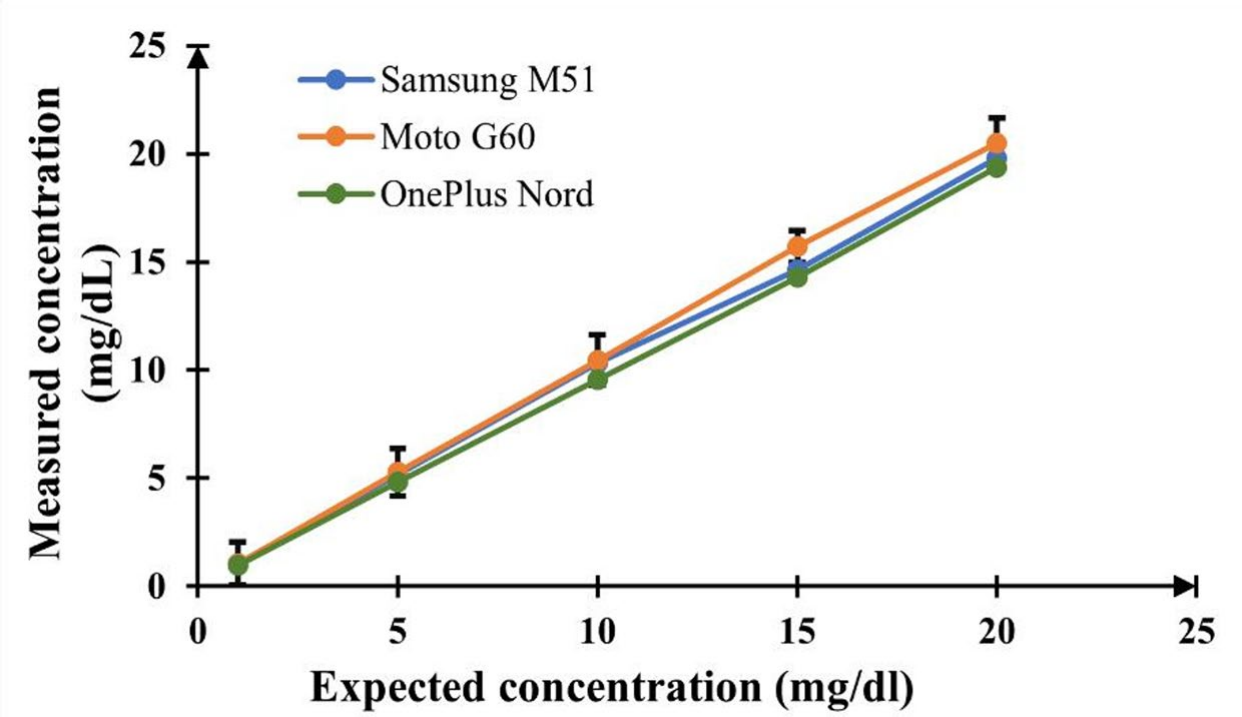
Comparison using three smartphones of the measured biomarker concentrations against expected concentrations.

Smartphone-based imaging shows variation in images due to differences in camera hardware, sensor sensitivity, and lens optics. The prediction accuracy of CNN models is reduced by these factors. This can be fixed by a software-based recalibration method that requires only a minimal number of reference standards. Two reference solutions: a water or blank (0 mg/dL) and a mid-range standard (10 mg/dL) of the target biomarker were used in the recalibration procedure. The predicted biomarker concentrations obtained from three different smartphone cameras (Samsung M51, Moto G60, and OnePlus Nord), which are compared to expected concentrations, are shown in Fig. 6. The average percentage deviation remained below 3% even with the hardware and software differences. This shows the robustness of the proposed recalibration approach.

### Validating the developed analyzer against commercial standards

To evaluate the real-world performance of the developed microfluidic sensing system, a comparative analysis was conducted with real samples against a gold-standard commercial biochemical analyzer. These measurements were selected in such a way that both the physiological and pathological ranges were used to assess the agreement between the predicted results of the deep learning-enabled device and the commercial reference system (Peerless Autolab Versa).

The comparative results is summarized in Table 3. This comparison is made between the concentration values obtained from both analyzers. The developed system strongly coordinates with the commercial analyzer. Minor variations between the readings can have fundamental differences in hardware calibration, optical principles for detection, and processing of algorithms. However, these deviations are within the defined range of Clinical Laboratory Improvement Amendments (CLIA). The total allowable error (TEa) for liver biomarkers is within ± 10% of the target concentration^21^.

The Pearson correlation coefficient ($\:r)$ was used to evaluate the agreement between the developed platform and the commercial clinical analyzer, calculated as $\:r=\frac{\sum\:({x}_{i}-\stackrel{\prime }{x})({y}_{i}-\stackrel{\prime }{y})}{\sqrt{\sum\:({x}_{i}-\stackrel{\prime }{x}{)}^{2}\sum\:({y}_{i}-\stackrel{\prime }{y}{)}^{2}}}$, where $\:{x}_{i}$and $\:{y}_{i}$are the measured values from the commercial and developed analyzers, respectively. The obtained correlation coefficients (*r* = 0.9994–0.9998) show excellent linearity and strong concordance across all tested biochemical parameters.

**Table 3:** Comparative analysis for liver function tests of a developed smartphone-based platform and a commercial analyzer.

Test	Analyzer	Results					*r*
Bilirubin direct mg/dl	Commercial	0.52	2.13	8.25	12.45	17.1	0.9997
	Developed	0.55	2.20	8.89	12.90	17.60	
	% Variation	5.77	3.29	7.76	3.61	2.92	
Bilirubin total mg/dl	Commercial	0.32	3.47	9.55	14.25	18.41	0.9994
	Developed	0.35	3.78	10.1	14.8	19.21	
	% Variation	9.37	8.93	5.76	3.86	4.35	
ALT U/L	Commercial	15.14	74.58	105.24	178.77	225.25	0.9998
	Developed	15.7	79.3	110	183.5	230	
	% Variation	3.70	6.33	4.52	2.65	2.11	
AST U/L	Commercial	20.15	62.25	124.47	166.66	280.24	0.9996
	Developed	21.78	66.9	129.2	171.4	284.9	
	% Variation	8.09	7.47	3.80	2.84	1.66	

The detection range for direct and total bilirubin was 0.1 to 20 mg/dL, and having limits of detection (LOD) to be 0.1 mg/dL and 0.05 mg/dL, respectively, for *n* = 5. The detection range extended from 10 to 300 U/L, with LODs of 2.97 U/L and 2.50 U/L, respectively, for *n* = 5 for ALT and AST.

The smartphone interface automatically gives a prompt advising a 1:5 dilution with reagent diluent or deionized water for samples exceeding the validated linearity range. The recalculated concentration is then calculated by simply multiplying the measured value by the dilution factor. The standard dilution practice used in pathology laboratories reflects these features and ensures accuracy even for samples with abnormally high enzyme or bilirubin levels.

### Repeatability analysis

**Fig. 7:**
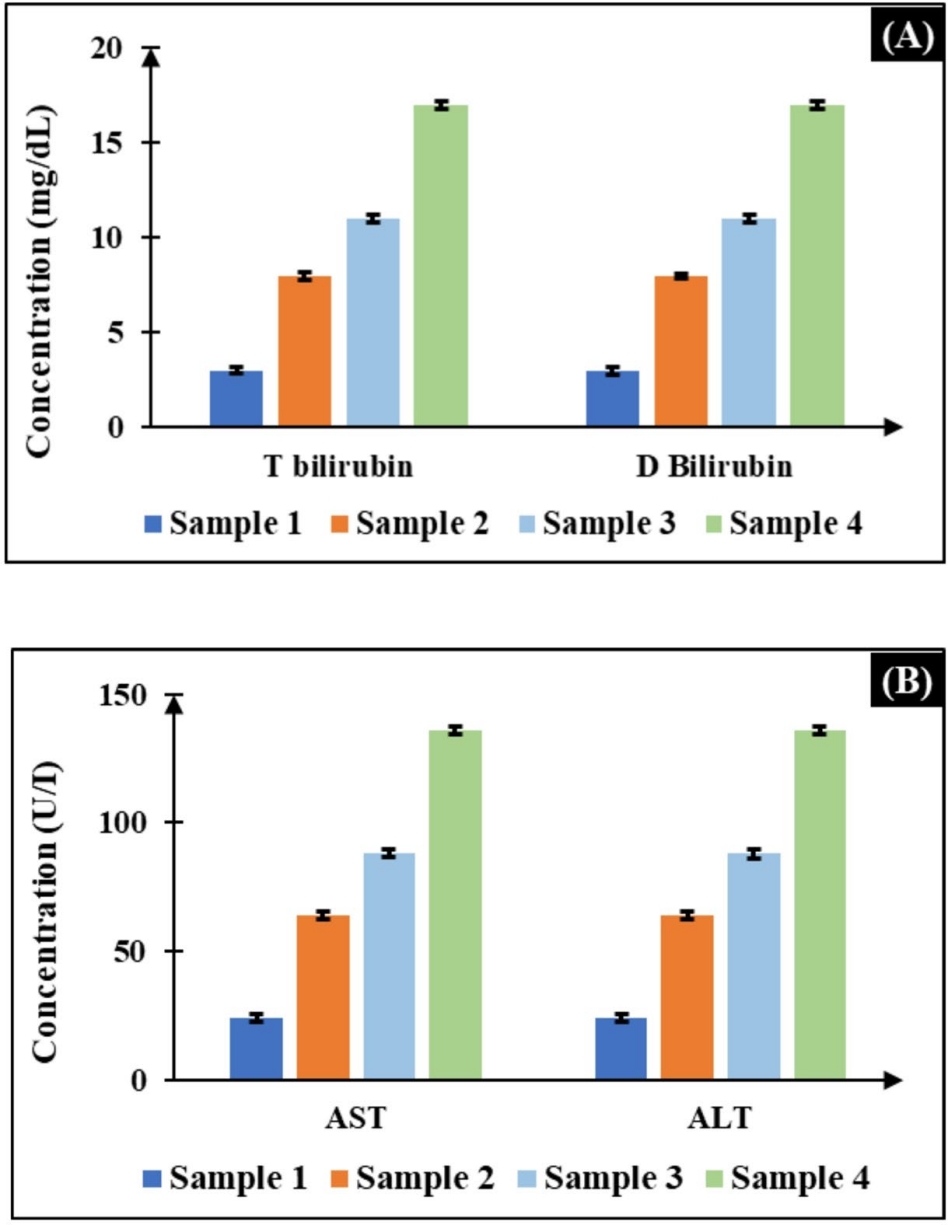
Repeatability analysis of consistent biomarker across multiple trials (**A**) for T Bilirubin and D Bilirubin, (**B**) for AST and ALT.

To guarantee the reliability of the developed biosensing platform for real-world applications, repeatability evaluation is important. For this purpose, multiple tests were conducted under different conditions, including repeated trials on the same day, testing on different days, and measurements by different users^22^. The developed system shows strong performance across all the factors. The %CV remained below 3% for all repeatability experiments. This indicates high precision in results as shown in Fig. 7A for T Bilirubin and D Bilirubin, and in Fig. 7B for AST and ALT. The system’s stability is validated by the low %CV values. These findings are further illustrated in the graphical results, which confirm that the developed platform offers high repeatability and reliability.

### Comparative analysis of existing technologies

A comparative analysis against recent portable biosensing platforms was performed as reported in the literature. The comparison focused on the range of detection, limit of detection (LOD), sample volume, detection methodology, and compatibility of the smartphone.

The existing solutions are limited to one or two liver function biomarkers and focus on total or direct bilirubin. Some methods include image analysis, nanomaterial-based fluorescence, chemiluminescence, or radiochemical sensing. While some platforms address enzymatic biomarkers like ALT or AST, they require larger sample volumes, lack mobility, or do not support multi-analyte detection within a single platform. The developed system is useful for detecting all four critical liver biomarkers across wide clinical ranges and with sub-clinical LODs.

The proposed system has the uniqueness to offer simultaneous analysis of all four liver biomarkers using only 5 µL of sample. A CNN-based regression model processes chromatic features from the microfluidic reactions, at the same time, the developed Android application ensures guided operation, real-time analysis, and result reporting. A detailed comparison with existing technologies is summarized in Table 4.

**Table 4:** Comparison of different parameters with existing technologies.

Analyte	Linear detection range	LOD	Accuracy	Sample volume	Detection techniques used	References
Bilirubin	0.5–7.0 mg/dL	0.48 mg/dL	R^2^ > 0.95	5 µL	Machine Learning	^23^
Glucose, Triglycerides, Urea	Variable	~ 0.01 mM	R^2^ = 0.94 − 0.99	10–20 µL	Machine Learning	^24^
Bilirubin	0.1–2.0 mg/dL	0.56 mg/dL	R^2^ = 0.931	0.1 mL	Image analysis	^25^
Bilirubin	0.18 µM to 14.29 µM	38.96 nM	–	20 µL	Nanomaterial-based fluorescence sensing	^26^
Bilirubin	1.2 to 950 µM	0.799 µM	–	40 µL	Image analysis, colorimetric assay	^27^
Bilirubin (Sclera)	–	–	R^2^ = 0.90	Image only	Per-Image Calibration, Two-Step Processing	^28^
Bilirubin	above 250 µmol/L	NA	R^2^ = 0.84	600 µL	Physics-based approach	^29^
Bilirubin	greater than 80 µmol/L	NA	R^2^ = 0.90	Image only	Image analysis	^30^
Bilirubin (Sclera)	less than 1.3 mg/dL	~ 0.3 mg/dL	R^2^ = 0.89	Image only	Machine Learning	^31^
Neonatal bilirubin	1.1 to 23.0 mg/dL.	1.1 mg/dL	R^2^ = 0.973	50 µL	Optical density measurements	^32^
AST	5 to 40 U/L	–	–	–	Digital Colorimeter	^33^
ALT	5 to 35 U/L					
AST ALT	1 µM to 0.5 mM	–	R^2^ = 0.998	–	Radiochemical Analysis	^18^
T Bilirubin	0.1 to 20 mg/dL	0.1 mg/dL	R^2^ = 0.997	5 µL	CNN with regression-based colorimetric shifts	This work
D Bilirubin	0.1 to 20 mg/dL	0.05 mg/dL				
ALT	10 to 300 U/L	2.97 U/L				
AST	10 to 300 U/L	2.5 U/L				

### Android application development

A Liver Sense Android application has been developed to ensure accurate testing, real-time analysis, and result visualization without any specialized hardware or professional expertise. Flutter is used to develop the frontend, which provides a user-friendly interface compatible with a number of Android devices for test selection, result viewing, and report generation. Firebase provides cross-platform app development for secure user authentication, session management, and data storage in Firestore for real-time use.

**Fig. 8:**
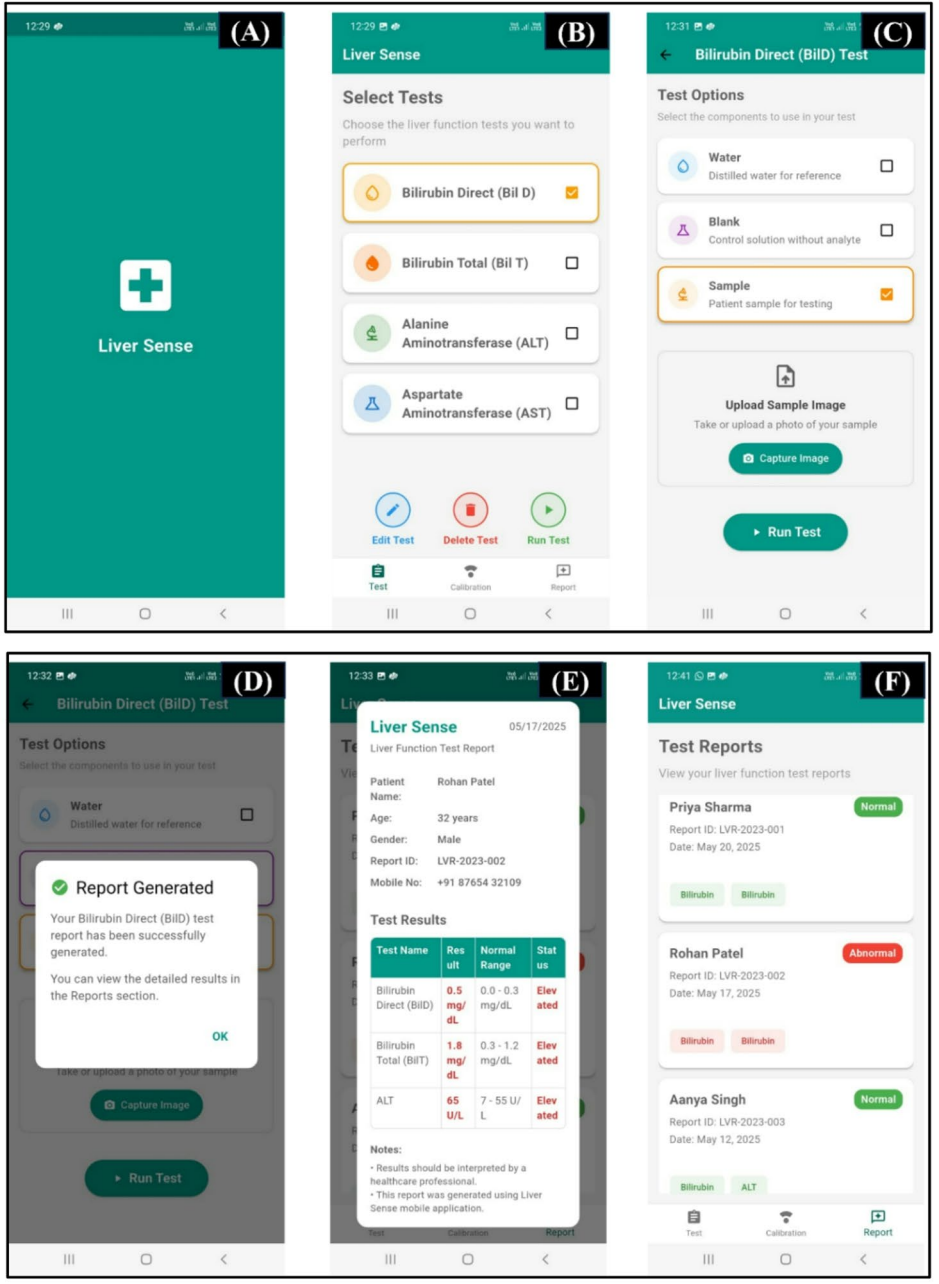
Graphical user interface of the Liver Sense mobile application for testing the liver function. (**A**) Home screen providing access to core functionalities. (**B**) Interface for choosing among four key liver biomarkers. (**C**) Test configuration screen for Direct Bilirubin test, having options for selecting sample type and to upload or capture test images. (**D**) Confirmation message showing proper test initiation. (**E**) Detailed report view that displays quantified biomarker results. (**F**) Summary dashboard listing multiple test reports for future tracking.

The backend is developed using FastAPI, it works as a RESTful API interface for executing test protocols, processing inputs given by users, and delivering biomarker predictions. It also acts as an API gateway, that manages microservices responsible for image preprocessing, model inference, and database communication. Modular scalability, reduced latency, and efficient handling of clinical test workflows are supported by this architecture^34^. This software-based approach eliminates dependency on external interfaces, making the system highly scalable, low-cost, and suitable for resource-constrained healthcare areas. The application’s user interface and key features are illustrated in Fig. 8A–F.

## Conclusion

This work presents a concise, smartphone-based liver function testing platform that is a combination of a stereolithography (SLA) 3D-printed microfluidic flow cell and deep learning-based image analysis for calculating total bilirubin, direct bilirubin, ALT, and AST. CNN-based regression models were used to evaluate Chromogenic reactions that are captured under controlled light conditions. It gave high prediction accuracy and comparable clinical detection limits across the complete physiological and pathological ranges. The smartphone camera was used for imaging and processing, which are of great use for point-of-care operations. Three smartphone models showed < 3% cross-device deviation. This was evident that the smartphone-based sensing approach gives laboratory-comparable performance, maintaining portability and scalability. The Android application allows real-time processing and an easy-to-understand layout of the displayed results. This reduces the need for any external hardware and training required for operating it. The system is designed to be low-cost, user-friendly, and easily deployable in decentralized healthcare environments. A gold-standard commercial analyzer and repeatability analysis across users and devices confirm the system’s accuracy and precision. This platform shows great progress toward liver function monitoring, with strong potential in community health programs, mobile clinics, and resource-constrained areas.

## Data Availability

The original contributions presented in the study are included in the article; further inquiries can be directed to the corresponding authors.
